# Supplementary material for: Structural Analysis of the Simultaneous Activation and Inhibition of γ-Secretase Activity in the Development of Drugs for Alzheimer’s Disease
Source: Pharmaceutics. 2021 Apr 8;13(4):514. doi: 10.3390/pharmaceutics13040514 (PMC8068388; doi:10.3390/pharmaceutics13040514)
Supplement: Supplementary file 1 [file pharmaceutics-13-00514-s001.zip › pharmaceutics-1143950 sup for xml.docx]

Supplementary Materials: Structural Analysis of Simultaneous Activation and Inhibition of γ-Secretase Activity in
Development of Drugs for Alzheimer’s disease

Željko M. Svedružić, Katarina Vrbnjak, Manuel Martinović and Vedran Miletić

Video S1. All-atom molecular dynamic studies of penetration of DAPT molecules into the active site tunnel. (Available online)

| **Citation: DAPT molecules into the active site tunnel, A.-a.m.d.s.o.p.o.** Željko M. Svedružić, Katarina Vrbnjak, Manuel Martinović and Vedran Miletić. *Pharmaceutics* **2021**, *13*, x. https://doi.org/10.3390/xxxxx  **Received: 26 February 2021**  **Accepted: 6 April 2021**  **Published: date**  **Publisher’s Note: MDPI stays neutral with regard to jurisdictional claims in published maps and institutional affiliations.**  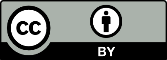  **Copyright: © 2021 by the authors. Submitted for possible open access publication under the terms and conditions of the Creative Commons Attribution (CC BY) license (http://creativecommons.org/licenses/by/4.0/).** |
| --- |

The presenilin structure is shown as a transparent white surface to show structures underneath the surface. The active site Asp 257 and Asp 358 are shown as red licorice, while the PAL motif (a.a. 433 to 435) is shown as black licorice. DAPT molecules are shown as green licorice. The Aph1 structure is shown as a transparent yellow surface. The boundaries of the lipid bilayer are illustrated by marking the surrounding water molecules with red dots. The simulation shows 200 nanoseconds of molecular events, one frame recorded every nanosecond. All four biphasic-drugs can penetrate in the active site tunnel to different depth with different rates (Supplementary Figure S3). We show penetration by DAPT to highlight how its flexible, elongated structure, with aromatic rings on each can best adapt to dynamic changes in protein structure.

| 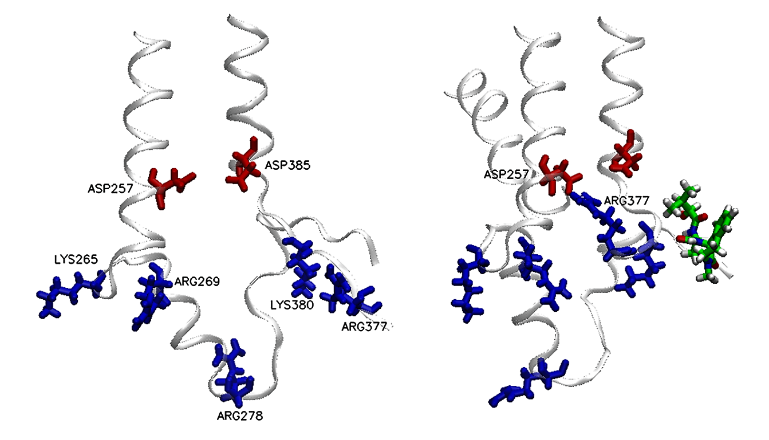 |
| --- |
| (**A**) |
| 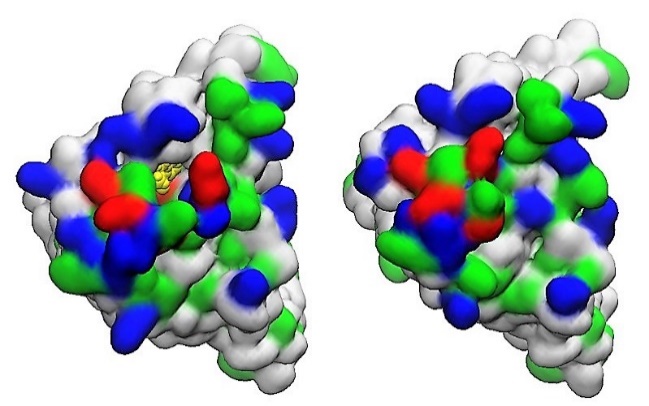 |
| (**B**) |

**Figure S1.** (**A,B**) Dynamic protein structures at the cytosolic end of the active site tunnel.

Molecular dynamic calculations showed that the TM6, TM6a, and TM7 sections at the cytosolic end of the active site tunnel are highly dynamic structures (Figure 3). Dynamic changes are driven by densely packed charged amino acids (Figure 4C), that complete for dynamic salt-bridges with the surrounding water molecules. (**A**) The structures illustrate 10.7 Å motion of positive Arg 377 towards the negative Asp 257 in the active site. Arg 377 can also form similar interactions with the negatively charged C-terminal on the nascent Aβ substrate or with the negatively charged 3-peptide by-products (Figure 3B). Such interactions can affect processive cleavages of the nascent Aβ catalytic intermediates. The semagacestat molecule is shown in green to show that drugs can affect these dynamics interactions. (B) The cytosolic end of presenilin structure is shown in surface mode to illustrate penetration of semagacestat molecule into the active site tunnel in the presence of Aβ46 (Figure 7). Semagacestat molecule is shown in yellow, while amino acids are shown as blue-positive, red-negative, green-polar not charged, and white-hydrophobic.

| 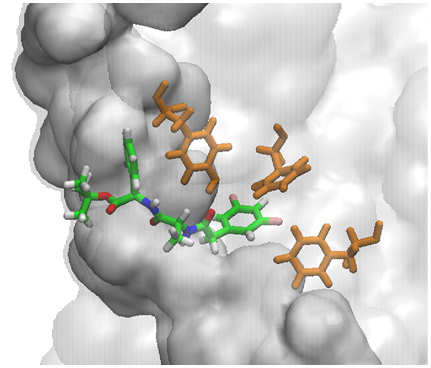 |
| --- |
| (**A**) |
| 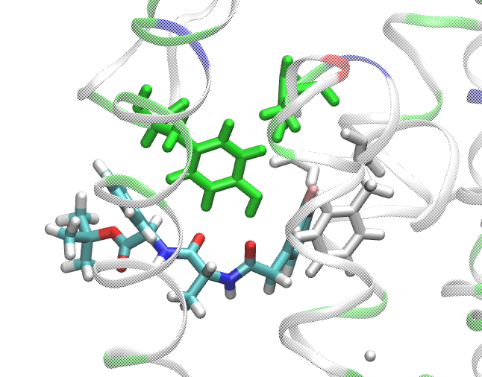 |
| (**B**) |
| 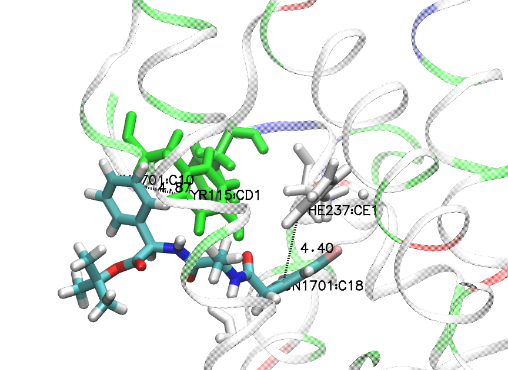 |
| (**C**) |

Figure S2. (A–C) Binding interactions for DAPT molecule at the membrane-embedded end of the active site tunnel with (A) and without the substrate (B–C).

**(A) The p**resenilin structure is shown as a gray transparent surface, while the substrate is shown as a gray opaque surface. DAPT molecule is shown in green, while the surrounding amino acids are shown as orange models. The substrate can block π-π stacking interactions between the drugs and the aromatic amino acids**. (B–C)** DAPT molecule in the absence of the substrate can penetrate in the position of the substrate where it can form π-π stacking interactions with the aromatic amino acids, most notably Phe 235 and Tyr 115.


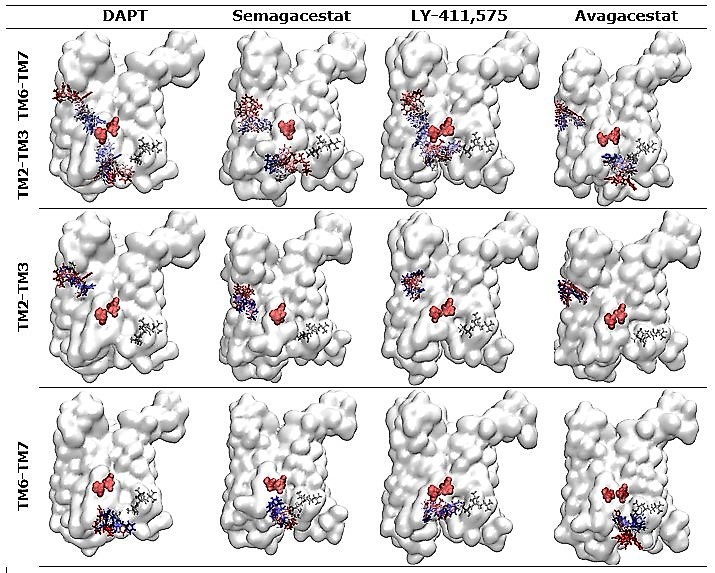


Figure S3. Drugs can cooperate in penetration into the active site tunnel on presenilin 1.

γ-Secretase structures with drugs bound at each end of the active site tunnel (TM2-TM3 and TM6-TM6a-TM7) have been compared with the structures that had drugs bound only to one of the two ends of the tunnel (TM2-TM3 or TM6-TM6a-TM7). The picture shows presenilin subunits as transparent surface, active site Asp257 and Asp385 as red VdW models, and PAL as gray licorice [[1](#_ENREF_1)]. The binding trajectories that represent 150 nanoseconds of molecular events are depicted showing drug structures at 0, 30, 60, 90, 120, and 150 nanoseconds colored in RWB scale respectively.

|  | **DAPT**  **0 Nanoseconds** | **DAPT**  **300 Nanoseconds** | | **Semagacestat**  **0 Nanoseconds** | | **Semagacestat**  **300 Nanoseconds** | |
| --- | --- | --- | --- | --- | --- | --- | --- |
| Membrane site | 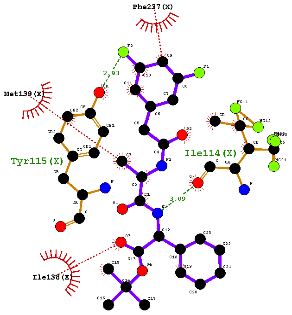 | 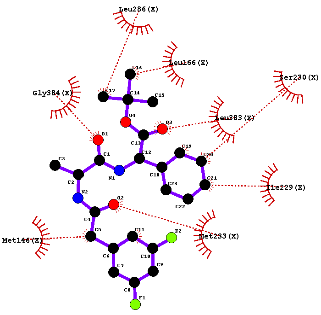 | | 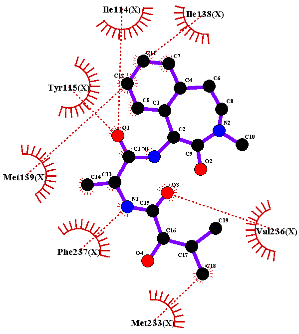 | | 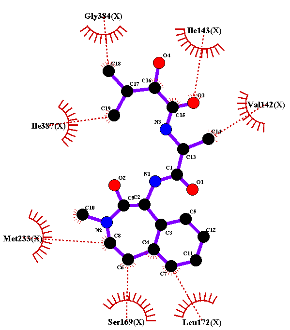 | |
| Cytosolic site | 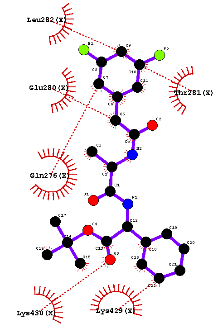 | 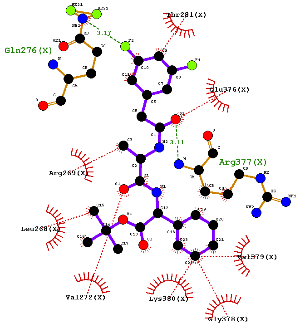 | | 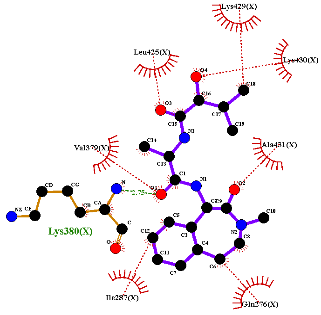 | | 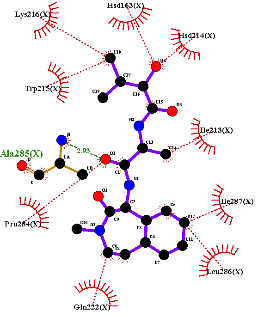 | |
| Aph1 site | 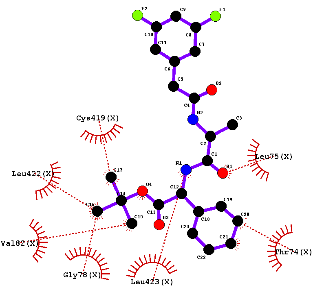 | 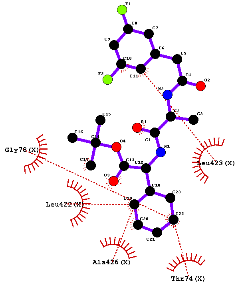 | | 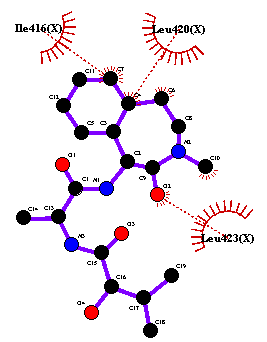 | | 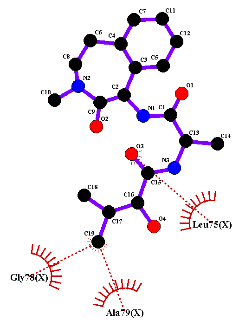 | |
|  | **LY-411,575**  **0 Nanoseconds** | | **LY-411,575**  **300 Nanoseconds** | | **Avagacestat**  **0 Nanoseconds** | | **Avagacestat**  **300 Nanoseconds** |
| Membrane site | 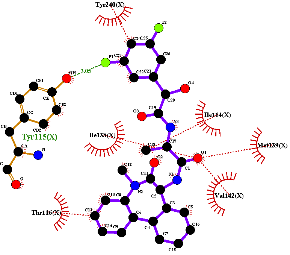 | | 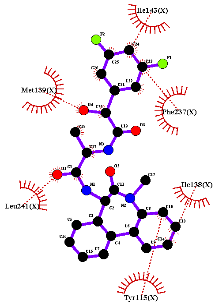 | | 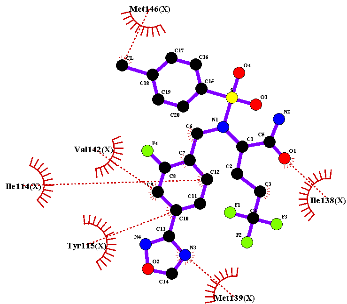 | | 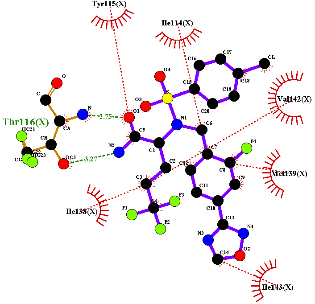 |
| Cytosolic site | 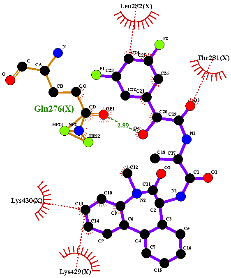 | | 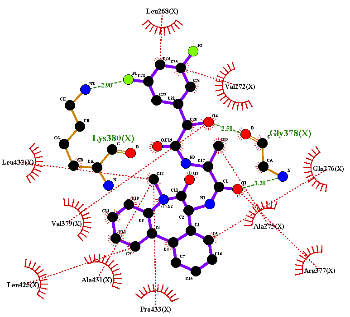 | | 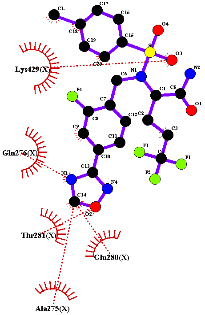 | | 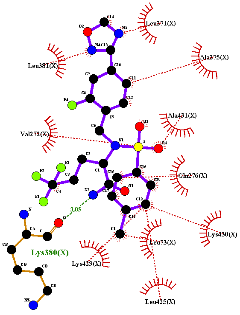 |
| Aph1 site | 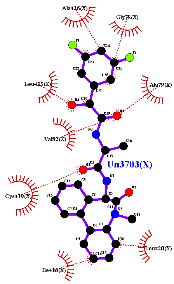 | | 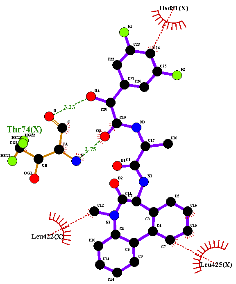 | | 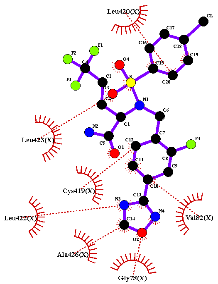 | | 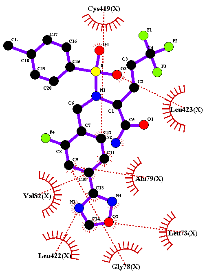 |

Figure S4. Binding interactions for the four drugs to with γ-secretase in the absence of the substrate (LigPlus, [[2](#_ENREF_2),[3](#_ENREF_3)]).

Dynamic changes in binding interactions are depicted by comparing binding contacts at the start, and the end, of molecular dynamics calculations that represent 300 nanoseconds of molecular events. All four drugs have binding sites at each end of the active site tunnel. The first site is the membrane-embedded entrance to the active site tunnel. The drugs form dynamic contacts with residues 113–116 (LIYT) on the loop between TM2 and TM3, with residues 138–142 (IMISV) on TM2, and depending on the depth of penetration with the residues around Phe237 on TM5. All four drugs have three major interaction sites at the cytosolic end of the active site tunnel. The drugs form at the start dynamic contacts with residues 276–280 (QERNE) at TM6a. Depending on the depth of penetration with residues 429–433 (KKALP) on the loop between TM8 and TM9, PAL motif (433–435), and the b-sheet residues 376–382 (ERGVKLG) at the endo-proteolytic site. At the Aph1 site, all four drugs have two major sites of interaction, residues 76–82 on TM1 and 419–425 on TM8, in parallel to the interactions with hydrophobic amino acids on Aph1 subunits (not shown). The Aph1 site is dominated by hydrophobic interactions and shows no places for possible polar or π-π stacking interactions. The polar interactions are marked with the green dashed line and the distances. All other interactions are hydrophobic and labeled with red dashed lines. The relative distances between the labels and the drug illustrate the relative distances in the three-dimensional structures. Carbon atoms are presented in black, oxygen in red, nitrogen in blue, and halogen in green as shown in Figure 2 in the main text.

|  | | **DAPT + Substrate**  **0 Nanoseconds** | | **DAPT + Substrate**  **300 Nanoseconds** | | **Semagacestat + Substrate**  **0 Nanoseconds** | | **Semagacestat + Substrate**  **300 Nanoseconds** |
| --- | --- | --- | --- | --- | --- | --- | --- | --- |
| Membrane site | | 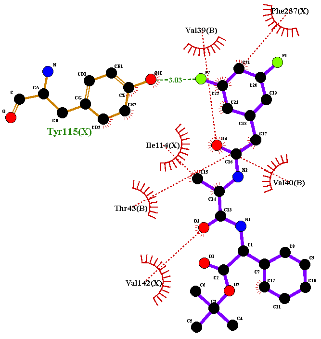 | | 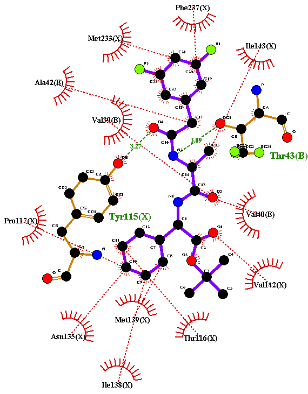 | | 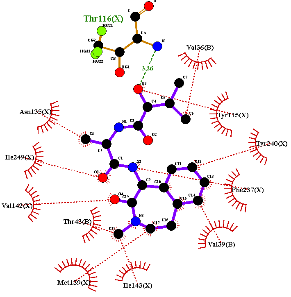 | | 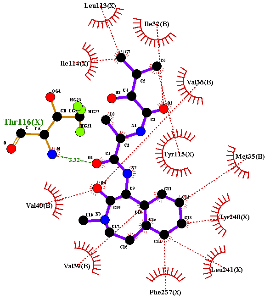 |
| Cytosolic site | | 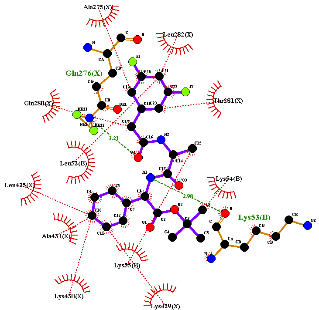 | | 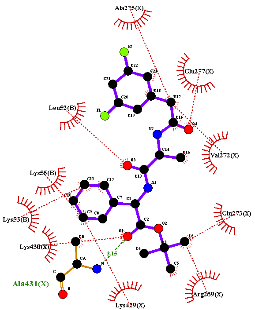 | | 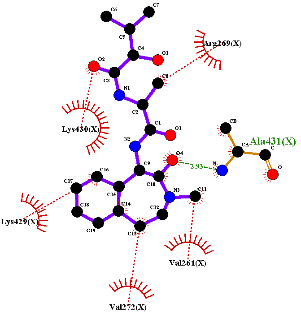 | | 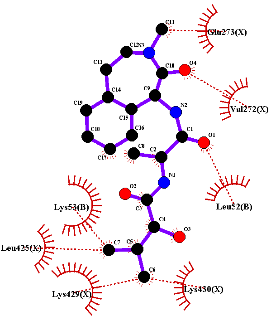 |
| Aph1 site | | 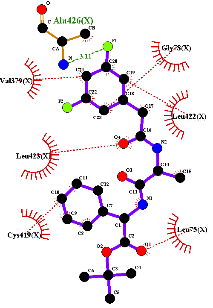 | | 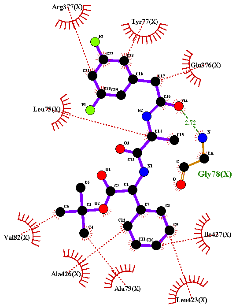 | | 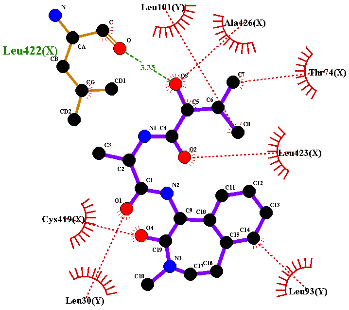 | | 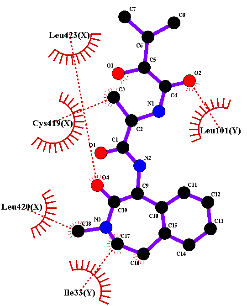 |
|  | **LY-411,575 + Substrate 0 Nanoseconds** | | **LY-411,575 + Substrate**  **300 Nanoseconds** | | **Avagacestat + Substrate**  **0 Nanoseconds** | | **Avagacestat + Substrate**  **300 Nanoseconds** | |
| Membrane site | 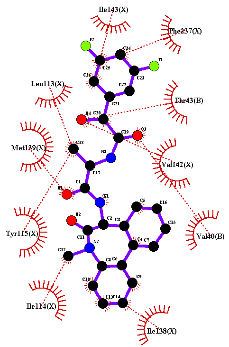 | | 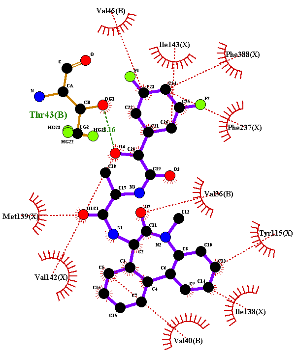 | | 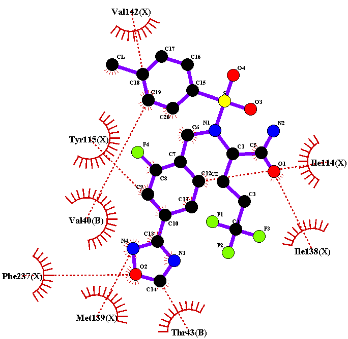 | | 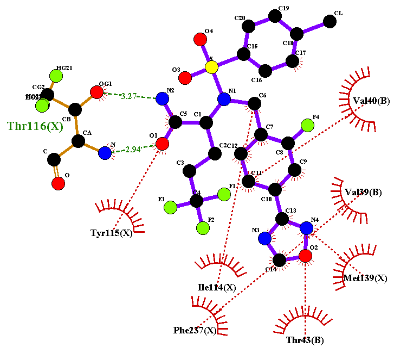 | |
| Cytosolic site | 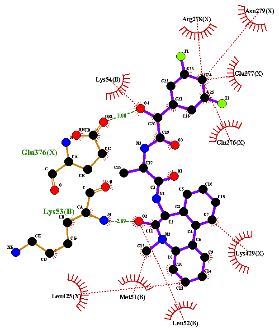 | | 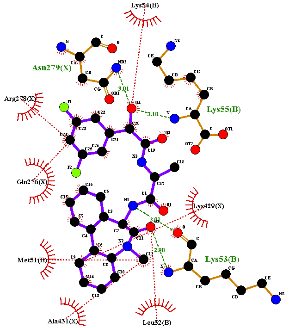 | | 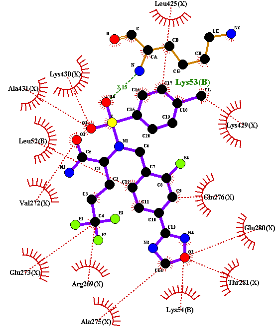 | | 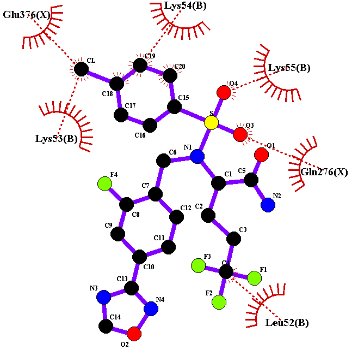 | |
| Aph1 site | 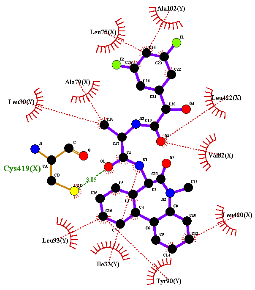 | | 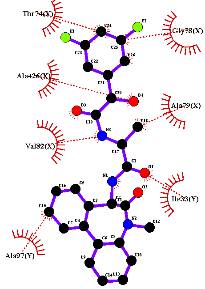 | | 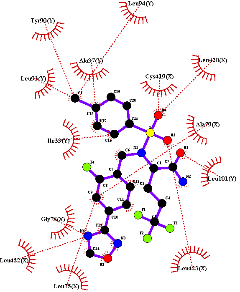 | | 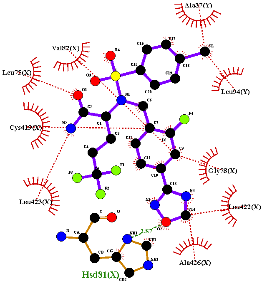 | |

Figure S5. Binding interactions between the four drugs and γ-secretase, when the enzyme is in complex with its amyloid substrate (LigPlus, [[2](#_ENREF_2),[3](#_ENREF_3)]).

Dynamic changes in binding interactions are depicted by comparing binding contacts at the start, and the end, of molecular dynamics calculations that represent 300 nanoseconds of molecular events. Amino acids in the presenilin subunit are labeled with X, in the Aph1 subunit with Y, and in the amyloid substrate with B. With all four drugs, the substrate has three effects on drug-presenilin interactions. The substrate can prevent the penetration of the drugs into the active site tunnel, the substrate forms strong polar interactions with the drug, and the substrate can displace some of the presenilin-drug interactions that are present in the absence of the substrate. At the membrane-embedded end of the active site tunnel, all four drugs interact with the substrate at the Val40 site, but occasionally the interactions can reach as far as Met 35 or as deep as Thr 43. The adjacent GXXXG dimerization domains are known to interact with the drugs, while the residues 43-46 are known as the sites with pathogenic mutations [[4](#_ENREF_4),[5](#_ENREF_5)]. At the cytosolic end of the active site tunnel, the drugs form polar interactions with the Lys-Leu rich domain, residues 51–55. The substrate has no significant effects on drugs bound at the Aph1 site. In all of the presented figures, the polar interactions are marked with the green dashed line and the distances. All other interactions are hydrophobic, including the π-π stacking interactions, and labeled with dashed red lines between the atoms and at the surface of the atoms. The relative distances between the labels and the drug structures illustrate their relative distances in the three-dimensional structures. Carbon atoms are presented in black, oxygen in red, nitrogen in blue, and halogen in green as shown in Figure 2 in the main text.

References

1. Sato, C.; Takagi, S.; Tomita, T.; Iwatsubo, T. The C-terminal PAL motif and transmembrane domain 9 of presenilin 1 are involved in the formation of the catalytic pore of the gamma-secretase. *J Neurosci* **2008**, *28*, 6264-6271.

2. Wallace, A.C.; Laskowski, R.A.; Thornton, J.M. LIGPLOT: a program to generate schematic diagrams of protein-ligand interactions. *Protein engineering* **1995**, *8*, 127-134, doi:10.1093/protein/8.2.127.

3. Zhou, R.; Yang, G.; Guo, X.; Zhou, Q.; Lei, J.; Shi, Y. Recognition of the amyloid precursor protein by human γ-secretase. *Science* **2019**, *363*, doi:10.1126/science.aaw0930.

4. Beel, A.J.; Mobley, C.K.; Kim, H.J.; Tian, F.; Hadziselimovic, A.; Jap, B.; Prestegard, J.H.; Sanders, C.R. Structural studies of the transmembrane C-terminal domain of the amyloid precursor protein (APP): does APP function as a cholesterol sensor? *Biochemistry* **2008**, *47*, 9428-9446.

5. Richter, L.; Munter, L.M.; Ness, J.; Hildebrand, P.W.; Dasari, M.; Unterreitmeier, S.; Bulic, B.; Beyermann, M.; Gust, R.; Reif, B., et al. Amyloid beta 42 peptide (Abeta42)-lowering compounds directly bind to Abeta and interfere with amyloid precursor protein (APP) transmembrane dimerization. *Proc Natl Acad Sci U S A* **2010**, *107*, 14597-14602.
